# Supplementary material for: Genome-Based Mexican Diet Bioactives Target Molecular Pathways in HBV, HCV, and MASLD: A Bioinformatic Approach for Liver Disease Prevention
Source: Int J Mol Sci. 2025 Sep 15;26(18):8977. doi: 10.3390/ijms26188977 (PMC12470150; doi:10.3390/ijms26188977)
Supplement: Supplementary file 1 [file ijms-26-08977-s001.zip › ijms-3750657-supplementary.pdf]

Table S1. Predominant Mexican Foods and Their Antiviral Nutrients: Key Dietary Components Against Hepatitis C and B."

Ingredients	Nutrient / Bioactive Compounds	Type of Study	Effect	Ref.
		**Hepatitis B**		
Grapes, plums, peanuts, apples, blueberries	Resveratrol	IVi / IVo	Inhibits HBV replication; reduces tumor volume in HBV-induced hepatocellular carcinoma; induces apoptosis.	[47]
Sunflower seeds, almonds, spinach, broccoli	Vitamin E	RCT	Reduces ALT levels; decreases HBV DNA viral load; suppresses pro-inflammatory cytokines.	[48,49]
Milk	Lactoferrin	IVi	Blocks HBV entry into hepatocytes; binds to HBsAg to inhibit activity.	[50,51]
Tuna, sardines, shrimp, turkey, rice	Selenium	CT	Activates p53 to suppress HBV transcription; reduces the risk of primary liver cancer.	[52]
Turmeric	Curcumin	IVi / IVo	Inhibits HBV transcription and replication; decreases cccDNA levels; suppresses NFκB-mediated inflammation.	[53,54]
Lettuce, Mexican oregano	Luteolin-7-O-glucoside	IVi	Reduces HBV RNA and DNA levels; inhibits HBsAg secretion; has antioxidant and immunomodulatory properties.	[55]
Moringa oleifera leaves	Moringa Extracts	IVi	Reduces cccDNA levels; inhibits NFκB and pro-inflammatory cytokines; offers antifibrotic effects.	[56,57]
Coffee	Chlorogenic Acid	IVi	Reduces HBsAg and HBeAg secretion; decreases liver fibrosis progression; inhibits HBV replication.	[58-60]
Green tea	Epigallocatechin-3-gallate	IVi	Prevents HBV entry by degrading NTCP receptors; induces autophagosomes to impair HBV replication.	[61-64]
		**Hepatitis C**		
Fish like salmon and carp and eggs.	Docosahexaenoic Acid (DHA)	IVi	Inhibits HCV replication, counteracts core protein-induced lipid alterations.	[65-66]
Fish (carp), eggs	Eicosapentaenoic Acid (EPA)	IVi	Reduces inflammation and inhibits HCV replication.	[65-66]

Cloves, oregano, blackberries	Gallic Acid	IVi	Decreases HCV expression through antioxidant activity.	[67]
Sunflower seeds, almonds	Vitamin E	RCT	Reduces ALT levels and inflammatory markers in HCV infections.	[68]
Liver (duck, veal), carrots, broccoli	Vitamin A	IVi	Enhances antiviral effects of interferons against HCV.	[69]
Fish (carp), eggs	Vitamin D3	PCT	Modulates interferon signaling to inhibit HCV replication.	[70,71]
Liver, pork	Vitamin B12	IVi	Inhibits HCV translation by targeting internal ribosome entry sites.	[72]
Marjoram, cumin, turmeric, beans, and beef	Iron	IVi	Inhibits HCV polymerase activity and suppresses subgenomic replication.	[73]
Agave, sesame seeds, beef ribs	Zinc	IVi	Reduces HCV replication through modulation of RNA synthesis.	[74}

Table S1. Effects of various nutrients and bioactive compounds derived from common food sources on the replication, transcription, and inflammatory responses associated with Hepatitis B Virus (HBV) and Hepatitis C Virus (HCV). The information highlights the antiviral, anti-inflammatory, and immunomodulatory properties of specific vitamins, minerals, polyphenols, and fatty acids, showcasing their potential roles as complementary strategies in managing viral hepatitis. Regarding study types, RCT, RC, and RCC denote randomized (controlled) trials, including crossover designs. RPC, RDBC, and RDB refer to randomized placebo-controlled or double-blind formats, while IVo, IVi, and IS signify in vivo, in vitro, and in silico experiments. RBCC, RBC, and RDBCC indicate single- or double-blind crossover trials. CVC is case vs. controls, CS is cross-sectional, Coh is cohort, and CO is a crossover clinical trial. PCT stands for prospective clinical trial; RDBP and RTBPC indicate randomized double- or triple-blind placebo-controlled studies; and MA signifies a meta-analysis.

Table S2. Bioactive Compounds and Biological effects of Traditional Mexican Foods to treat and prevent metabolic dysfunction associated diseases.

Mexican Ingredient	Nutrient / Bioactive components	Type of Study	Effect	Ref.
Jicama *Pachyrhizus erosus L.*	Inulin, soluble and insoluble fiber, and Fructo-oligosaccharides	IVo	Insulin-Sensitizing: ↓Serum Insulin, HOMA-IR; ↑QUICKI. Hepatoprotective: ↓PEPCK, G6pase; ↑Hepatic Glycogen Hypoglycemic: ↓Serum glucose, GTT, Serum glucose, HbA1c; ↑ PM AMPK-AS160-GLUT4 pathway Anthropometric Improvement: ↓BW, WAT, BAT Anorexigenic: ↓ Food intake	[75-76]
Cucurbita maxima, Helianthus annuus, Linum usitatissimum (Pumpkin, Sunflower Seeds, Flaxseeds	EPA, ALA, DHA and Dietary Fiber	CS / CVC	Insulin-Sensitizing: ↓Insulin, HOMA-IR.<br>Lipid-Lowering: ↓C-LDL; ↑ C-HDL<br>Anti-inflammatory: ↓TNFα, IL-6, hs-CRP	[77]
Carya Illinoensis (Pecan)	MUFA, and phenolic compounds (Tanines, ellagic acid, urolitin)	RBCC	Insulin-Sensitizing: ↓ Serum insulin, HOMA-IR; ↑ HOMA-β. Lipid-Lowering: ↓ C-LDL; ↑ HDL2, HDL3, ApoAI, EPA, DHA	[78]
Psidium guajava (Guava)	Phenolic compounds (Kojic acid and 5-hydroxymethylfurfural)	RDBP	Insulin-Sensitizing: ↓ Insulin Secretion<br>Hypoglycemic: ↓Postprandial glucose	[79]
Portulaca oleracea (Purslane)	Polysaccharides, Flavonoids, ALA.	RDBC	Insulin Sensitizing: ↓ HOMA-IR. Hepatoprotective: ↓ALT, AST. Lipid-Lowering: ↓ LDL-C, TG; ↑ HDL-C<br>Hypoglycemic: ↓ FBG<br>Anthropometric improvement: ↓ BW, BMI, WC	[80]
Amaranth leaves (Quilitl)	Amaranth Hydrolysates, flavonoids, phenolic acids, anthocyanins, tannins, and phytosterols, and polyphenols.	IVo / IVi	Hypoglycemic: ↓ Postprandial glycemia, Hemoglobin glycation; ↑inhibition of α-glucosidase Antioxidant: ↓ABTS, DPPH, FeCl3, H2O2 radical scavenging; ↑Antioxidant capacity	[80-82]
Opuntia Ficus Indica, (Nopal) *Theobroma cacao* (Cacao) and crickets	Pectins and phenolic compounds.	IVo	Insulin-Sensitizing: ↓ Insulin, Resistin<br>Lipid-Lowering: ↓TC<br>Hypoglycemic: ↓Glucose<br>Anthropometric improvement: ↓ BW, %BF, VF	[83-85]

Opuntia Robusta fruit (prickly pear fruit)	Betacyanines and betalain	IVo	Hepatoprotective: ↓ AST, ALT, Caspase-3 activity<br>Anticarcinogenic: ↑ P53 liver expression<br>Antioxidant: ↓ ROS; ↑ MnSOD	[86]
Opuntia cochenillifera (Nopal Cladodes)	Phenolic compounds, and pectins.	IVo	Hypoglycemic: ↓ Glucose, α-glucosidase activity, glucose absorption<br>Antioxidant: ↓ DPPH scavenging activity	[87]
Theobroma Cacao (Cacao)	Polyphenols (Procyanidins and epicatechins), flavonoids	RCC / IVi	Insulin-Sensitizing: ↑ GLP-1 expression, insulin secretion<br>Hypoglycemic: ↓ Post prandial Glucose<br>Antioxidant: ↓DPPH	[85,88]
Zea Mays (White and Blue corn)	Polyphenols, Anthocyanins (cyanidin, pelargonidin, and peonidin), anthocyanidins (Cyanindin 3 -glucoside) phenolic compounds, ZeinH, insoluble fiber	IVo / IS/ IVi	Insulin-Sensitizing: ↑ Insulin Secretion, GIP; ↓Serum Insulin levels<br>Hepatoprotective: ↓ Liver weight, liver steatosis and inflammation<br>Lipid-Lowering: ↓TG, TC, LDL-C<br>Hypoglycemic: ↓ Glucose, IPGTT, GLP-1, OGTT<br>Antioxidant: ↓ MDA, FRAP; ↑ liver Sod1	[89-94]
Phaseolus Vulgaris (Beans)	Insoluble fiber, Anthocyanins (C3G-P, D3G and CF-P), lectins, α-amylase inhibitors (a-AI), phytohaemagglutinin and arcelins	IVi /IS/ RDBC	Insulin-Sensitizing: ↓Serum Insulin levels, C-peptide; ↑ Insulin secretion in pancreatic β-cells<br>Lipid-Lowering: ↓Ectopic fat accumulation, TC, LDL-C<br>Hypoglycemic: ↓ Serum glucose, IPGTT, GLP-1, OGTT, Glucose intestinal transport<br>Anthropometric improvement: ↓BW, WC<br>Anorexigenic: ↓ Ghrelin, desire to eat	[90,91, 95-97]
Persea Americana (Avocado)	MUFA, Manganese, Potassium, Folate, phytosterols, soluble and insoluble fiber, perseitol, avocatin B, Lutein ,α-carotene	RCC / IVo / RCT / RDBC	Insulin-Sensitizing: ↓ Insulin secretion,<br>HOMA-IR; ↑ PHD activity, AKT phosphorylation, glucose utilization in pancreatic and muscle tissue.<br>Lipid-Lowering: ↓sdLDL, CETP activity, TC, LDL-C, sdLDL-C, ApoB100; ↑ HDL-C<br>Hypoglycemic: ↓ Glucose<br>Antioxidant: ↓ LDL-ox and Mitochondrial oxidative stress<br>Anthropometric improvement: ↓BW, lipid accumulation, mesenteric fat	[98-100, 115,116]

Salvia Hispanica L. (Chia seeds)	ALA, fiber, phenolic compounds (quercetin and myricetin)	IVo / RDBC	Insulin-Sensitizing: ↑ IRS-1, Adiponectin secretion ↓ pAMPK/AMPK<br>Hypoglycemic: ↑ GLUT-4 in HT<br>Anti-inflammatory: ↓ hs-CRP<br>Antioxidant: ↓DPPH, ABTS<br>Anthropometric Improvement: ↓BF%, WC<br>Anorexigenic: ↓ Ghrelin	[101--103]
Capsicum spp. (Chili)	Capsaicin	IVi/ CO	Insulin-Sensitizing: ↑ Insulin secretion.<br>Hypoglycemic: ↓ OGGT; ↑Glucose uptake in MT through the activation of AMPK and p38 MAPK	[104-107]
Agave Tequilana Weber Var. Azul (Agave)	Agave Fructans (FOS and GOS), Inulin, Polyphenols	RCT / RPC / RDB / IVo	Lipid-Lowering: ↓TG, TC, LP, LDL-C, and ↑HDL-C<br>Hypoglycemic: ↓Serum Glucose<br>Anthropometric Improvement: ↓BW, BF%<br>Prebiotic: ↑*Bifidobacterium spp*	[108-111]
Arachis hypogaea (Peanut)	Arginine and MUFA	RBCC	Lipid Lowering: ↓LDL-C, TC, ↑ HDL-C	[112]
Ananas Comosus (Pineapple)	Anthocyanins, Hydroxycinnamic acids, and flavonols.	RCC	Antioxidant: ↑ NEAC, ↓ UA, SH production	[113]
Carica papaya (Papaya) NA	Ascorbic acid, protocatechuic acid Hexoxide, manghaslin, quercetin 3-Orutinoside, phenolic acids, caffeoyl hexoside, ferulic acid, β-cryptoxanthin,<br>β-carotene, lutein and zeaxanthin	IVi	Antioxidant: ↓DPPH, ↑FRAP	[114]
Solanum Lycopersicum (Tomato)	Dietary Fiber, Lycopene and β-Carotene	IE / RCC / CVC	Insulin-Sensitizing: ↓FIRI, serum insulin.<br>Lipid-Lowering: ↓TG, TC, LDL, PPARγ, C/EBPβ, C/EBPα; ↑LPL<br>Hypoglycemic: ↓Serum Glucose, IGP<br>Anti-inflammatory: ↓ TNFα	[117-119]

Table S2.- TG, TC, and LP represent triglycerides, total cholesterol, and lipoproteins, respectively. C3G-P refers to cyanidin-3-O-glucoside from purple corn, and CF-P to its semi-purified condensed forms. ADMA is asymmetric dimethylarginine; FOS and GOS are fructo- and galactooligosaccharides; WAT denotes white adipose tissue; and FFA indicates free fatty acids. C/EBP is the CCAAT/enhancer-binding protein; MDA stands for malondialdehyde; GTT is the glucose tolerance test; HbA1c is hemoglobin A1C; and IGP is the incremental glucose peak. TAC represents total antioxidant capacity, NEAC is non-enzymatic antioxidant activity, SH stands for thiols, UA for uric acid, PM for peripheral muscle, PTP for protein tyrosine phosphatase, and HT for heart tissue. Regarding study types, RCT, RC, and RCC denote randomized (controlled) trials, including crossover designs. RPC, RDBC, and RDB refer to randomized placebo-

controlled or double-blind formats, while IVo, IVi, and IS signify in vivo, in vitro, and in silico experiments. RBCC, RBC, and RDBCC indicate single- or double-blind crossover trials. CVC is case vs. controls, CS is cross-sectional, Coh is cohort, and CO is a crossover clinical trial. PCT stands for prospective clinical trial; RDBP and RTBPC indicate randomized double- or triple-blind placebo-controlled studies; and MA signifies a meta-analysis.
